# ILC-You in the Thymus: A Fresh Look at Innate Lymphoid Cell Development

**DOI:** 10.3389/fimmu.2021.681110

**Published:** 2021-05-06

**Authors:** Samuel B. Shin, Kelly M. McNagny

**Affiliations:** ^1^ Department of Experimental Medicine, University of British Columbia, Vancouver, BC, Canada; ^2^ School of Biomedical Engineering, University of British Columbia, Vancouver, BC, Canada; ^3^ Department of Medical Genetics, University of British Columbia, Vancouver, BC, Canada

**Keywords:** T-cell development, innate lymphoid cell development, layered hematopoiesis, neonatal, fetal, origins of lymphocytes

## Abstract

The discovery of innate lymphoid cells (ILCs) has revolutionized our understanding of innate immunity and immune cell interactions at epithelial barrier sites. Their presence and maintenance are critical for modulating immune homeostasis, responding to injury or infection, and repairing damaged tissues. To date, ILCs have been defined by a set of transcription factors, surface antigens and cytokines, and their functions resemble those of three major classes of helper T cell subsets, Th1, Th2 and Th17. Despite this, the lack of antigen-specific surface receptors and the notion that ILCs can develop in the absence of the thymic niche have clearly set them apart from the T-cell lineage and promulgated a dogma that ILCs develop directly from progenitors in the bone marrow. Interestingly however, emerging studies have challenged the BM-centric view of adult ILC development and suggest that ILCs could arise neonatally from developing T cell progenitors. In this review, we discuss ILC development in parallel to T-cell development and summarize key findings that support a T-cell-centric view of ILC ontogeny.

## Introduction

While hints of innate type immune cell subsets, including NK cells and lymphoid tissue inducer (LTi) cells, were discovered as early as 1970s and early 2000s respectively (1, 2), a more detailed and full characterization of the innate lymphoid cell (ILC) family emerged in the late 2000s (3). Undoubtedly, their classification marks a formative breakthrough that changed our perception of the immune system and immune homeostasis (4–6). In just over a decade, ILCs were shown to be important in allergic disease, autoinflammation and immune tolerance (7, 8). Previous and on-going studies have highlighted them as key drivers of inflammation and fibrosis in inflammatory bowel disease, inducers of chronic airway inflammation, and active players in other disorders such as obesity and cancer (9–14). In general, ILCs are tissue-resident and are triggered through relatively broad spectrum receptors for pathogens or inflammatory cues rather than specific-antigen receptors (BCRs or TCRs). Upon appropriate alarmin signaling, they orchestrate downstream responses by communicating with neighboring stromal and immune cells to adjust the cytokine microenvironment in a fashion that promotes protection, health and homeostasis at mucosal barrier sites (5, 15). In addition, they have also emerged as regulators of homeostasis and tissue repair in non-barrier organs (16).

Molecularly, ILCs are extremely heterogenous but, for convenience, have been grouped into subsets that resemble the classification of T cells based on their surface marker, cytokine and transcription factor profiles during development and activation (ILC1/NK, ILC2 and ILC3/LTi) (17, 18). Thus, like type 1, 2 and 17 helper T cells, ILC1, 2 and 3 are categorized according to the class of immune response they invoke upon perturbation. ILC1s include conventional NK cells and “helper ILC1s” and are defined by the production of interferon γ (IFN-γ) in response to IL-12, IL-15 and IL-18 (19). The transcription factor, T-bet, functions as their master regulator with Eomes being present in NK cells and a small subset of ILC1s (20, 21). ILC2s, on the other hand, depend on the expression of GATA3, and are responsible for generating type 2 cytokines such as IL-5, IL-9, IL-13 and amphiregulin upon stimulation by the alarmins, IL-25, IL-33 and TSLP (12, 22, 23). Lastly, ILC3s are defined by production of IL-17 and IL-22 in response to IL-23 and IL-1β signaling and are maintained by the transcription factors RORγt and RORα (24, 25). Within the ILC3 group, there also exists an LTi family that arises during embryogenesis and facilitate the formation of secondary lymphoid tissues (26). Broadly speaking, ILC1s are involved in the clearance of intracellular pathogens, ILC2s are associated with helminth infection and chronic airway inflammation in response to allergens (27), and ILC3s are predominantly implicated in gut immunity and responsible for establishing tolerance and mucus secretion (17).

Because ILCs closely resemble helper T cells, they are often regarded as the innate counterparts of Th1, Th2 and Th17 cells. Despite this, current dogma suggests that the ontogeny of ILCs and T cells are separate and distinct, and that the thymic microenvironment is dispensable for ILC maturation (19). This concept arose from three distinct observations made from seminal studies that identified ILCs and their function. First, unlike T cells, all subset of ILCs do not depend on the expression of surface antigen-specific T-cell receptors (TCR) for their development and activation (18). Therefore the thymus, the site which provides the appropriate niche for TCR gene rearrangements and signaling through TCR and other co-receptors, was not considered to be important in ILC biology. Second, they are present, expanded and functional in mice with profound lesions in T-cell development including *Foxn^nu/nu^* (nude) and in *Rag^-/-^* mice (28). Lastly, the characterization of early ILC progenitors in the adult BM and their restricted T-cell potential as they differentiate downstream of common lymphoid progenitors (CLP) have left an impression that the unique microenvironment of the thymus is dispensable for ILC development (29–32). As a result, investigations of ILC development in adult animals have focused predominantly on the key progenitor populations originally described in the adult BM, and the concept of ILCs arising during early T-cell development, until recently, has remained largely unexplored.

With the identification of BM ILC progenitors, the mapping of signaling pathways and factors that contribute to ILC development became the topic of intense investigation. Interestingly, these studies found that although ILCs do not undergo TCR-dependent development or activation, the factors required for their commitment and maturation are virtually indistinguishable from those found in T-cell development with the noteworthy exception of NFIL3 expression in early BM ILC progenitors (18, 32–34). Furthermore, recent reports have highlighted the ontogeny and presence of ILCs in embryonic/neonatal thymi (35, 36), suggesting that the bifurcation of T- and ILC-driven lineages, at least during neonatal life, is at the committed T-cell progenitor stage in the thymus. Here, we revisit ILC development in the context of neonatal T-cell development with a focus on recent literature highlighting layered ontogeny of ILC2s and TCR gene rearrangements and propose a model that more closely aligns the development of ILC and T-cell lineages during neonatal life.

## An Overview of the BM Model of ILC Development

**Figure 1:**
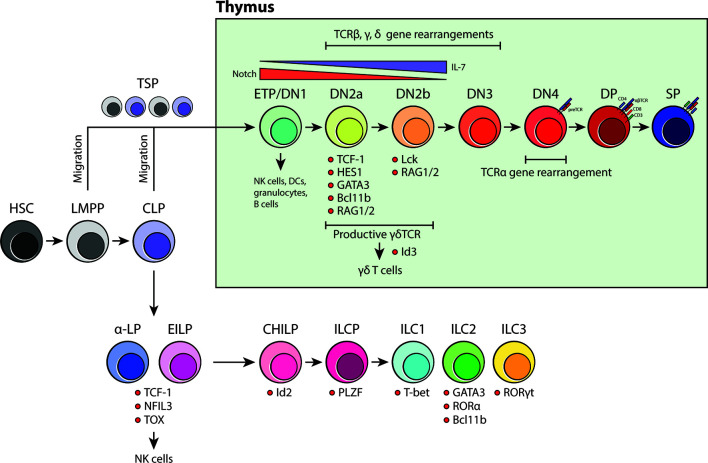
An overview of BM-dependent ILC and thymus-dependent T-cell development. Current models for both ILC and T-cell development suggest that they are of distinct lineages and that the site for maturation do not overlap beyond the CLP stage. ILCs have been proposed to develop in the BM whereas committed T-cell progenitors undergo intense development processes that are heavily influenced by the thymic niche.

The current framework of ILC development was built on the identification of CXCR6^+^ α-lymphoid progenitors (α-LP), early innate lymphoid progenitors (EILP), common helper ILC progenitors (CHILP) and ILC precursors (ILCP) in the adult BM (**Figure 1**). These were discovered and classified through a series of differentiation assays and transcription factor analyses that determined whether these precursors could competently become either ILC1, ILC2 or ILC3 (37). Briefly, it is thought that ILC lineage commitment occurs at the level of CXCR6^+^ α-LPs and EILPs. These progenitors are direct descendants of CLPs in that they retain both ILC and NK cell potential, but lack the ability to become functional T or B cells. Transcriptomic and protein analyses revealed that transcription factors NFIL3, TOX and TCF-1 (*Tcf7*) are critical in ILC lineage specification as they precede the expression of downstream ILC progenitor genes (29, 32, 38). In contrast, CHILPs and ILCPs represent further restricted downstream progenitors that have lost the ability to form T, B and NK cells. CHILPs and ILCPs are defined by the expression of transcription factors Id2 and PLZF, which reaffirms their commitment to helper ILC subsets (30, 39). Subsequently, based on the upregulated expression of T-bet (ILC1), GATA3, RORα, Bcl11b (ILC2) and RORγt (ILC3), the precursors become one of three mature ILC subsets (18) (**Figure 1**). Using this model as a foundation, later studies examined other possible sources of tissue-resident ILCs in adult and during embryonic development. Similar to the adult BM, it was shown that ILC development occurs in the fetal liver from Id2, PLZF and Arginase-1 (Arg-1) expressing ILC progenitors (40, 41). Fetal ILC development contributes predominantly to the generation of CD4^+^ LTi cells and NKp46^+^ ILC3s which then migrate to the gut and guide Peyer’s patch development (42). NK1.1^+^ ILC1 precursors are also found in the fetal liver. However, their gene expression signature resembles that of adult hepatic ILC1s suggesting that, perhaps, they represent liver-specific precursors that are long-lived, maintained, and self-renewed after their genesis in early liver development (43, 44). To date, the exact timepoint in which ILC progenitors colonize the periphery is unclear. Surprisingly, elegant recent parabiosis and pulse-label lineage tracing studies showed that the majority of tissue-resident ILC1, ILC2, ILC3 and LTi cells residing in the mucosal barrier tissues are not replenished through steady-state BM lymphopoiesis but instead self-renewed locally in their tissue of residence (44–47). Furthermore, tissue-resident lung Il18r1^+^ST2^-^ ILC2 progenitors have been recently shown to produce ILC2s locally upon immune challenge and independent of *de novo* production of ILCs in the BM (48). These simple observations then call into question the concept and significance of BM ILC generation and its contribution to the peripheral ILC pool.

## Normal Thymic Development of T Cells

In contrast to most other hematopoietic lineages, T-cell development is critically dependent on the ability of developing thymocytes to undergo a strict maturation process in the thymic microenvironment before colonizing the peripheral tissues (**Figure 1**). Originally, CLPs were thought to be the only branchpoint in which T-cell fate restriction occurs. However, with the discovery of lymphoid-primed multipotent progenitors (LMPP), the classical view of T-cell development was revised to include a secondary pathway that was independent of CLPs (49). LMPPs are defined by their expression of the fms-like tyrosine kinase 3 (Flt3) and are positioned upstream of CLPs in the differentiation hierarchy (50, 51). Like CLPs, they are restricted to the T-, B-cell and NK cell lineage, but are different in that they retain the potential for granulocyte/monocyte development. It has been shown that LMPPs are much more akin to ETPs in the thymus, suggesting that the thymus-seeding progenitor (TSP) pool includes both LMPPs and downstream CLPs lacking Ly6D^-^ expression (49, 52). Once in the thymus, CD4^-^CD8^-^ TSPs enter the double negative (DN) stage in T-cell specification. This stage is divided into four major compartments: DN1/ETP to DN4 (**Figure 1**). Depending on the differential expression of CD24, CD25, CD44 and CD117 (KIT) and the state of the TCR loci, developing DN thymocytes are classified as either DN1/ETP, DN2a, DN2b, DN3 or DN4 (49, 53). DN1 cells represent 0.01% of the total T-cell progenitors in the thymus and are surprisingly multipotent as they retain the ability to differentiate into cells of the myeloid and lymphoid lineages. However, upon arrival at the corticomedullary junction, Notch signaling induces a genetic program that secures their commitment to the T-cell fate and prepares them for TCR gene rearrangement (49). As these cells progress to the DN2 stage, they become localized within the subcapsular zone of the thymic cortex. Here, they begin rearranging their TCRβ, γ and δ loci *via* the activation of *Rag1* and *Rag2* genes. It is expected that DN2 cells become more dependent on IL-7 produced by thymic epithelial cells (TEC) as it is vital for their proliferation, survival and differentiation (54). There are two subtypes of DN2 cells, DN2a and DN2b, and they are characterized based on the expression of lymphocyte-specific protein tyrosine kinase (Lck) and the ability to suppress NK, myeloid and dendritic cell (DC) potential. Although they are more restricted than DN1/ETPs, DN2a cells are still relatively fluid in terms of their differentiation potential. This however, is lost as they continue through the process of TCR gene rearrangement and transition towards DN2b (55). At DN3, developing thymocytes extensively rearrange their DNA at the TCRβ, γ and δ loci and are selected for survival based on the expression of functional γδ or preTα/β (preTCR) chains. γδTCR expression, along with transcription factor Id3, promotes γδ T-cell development whereas preTCR expression guides the remaining DN3 cells to enter the DN4 stage. Once at the DN4 stage, thymocytes begin migrating back towards the medulla and initiate TCRα gene rearrangements upon preTCR signaling. After the formation of a functional αβTCR, thymocytes then upregulate CD4 and CD8 co-receptors to become double positive (DP) thymocytes. From here, DP cells are positively selected for reactivity with MHC, becoming either CD4 or CD8 single positive (SP) cells. Shortly thereafter, the surviving SP cells undergo negative selection against autoreactivity and become mature naïve T cells (49, 53, 54) (**Figure 1**).

## ILCs Are Long Lived Tissue-Resident Cells in Adult and Colonize Tissues During Fetal/Neonatal Development

The working model of adult hematopoiesis argues that the BM is responsible for generating and replenishing all blood and immune cells required for the lifetime an individual. Although this is true for some short-lived circulating leukocytes (56), it has become increasingly clear that tissue-resident cells such ILCs, macrophages, mast cell subsets and γδ T cells persist throughout life and, for the most part, expand and contract locally in their tissue of residence, largely independent of hematopoietic activity in the BM (45, 57–60). As outlined in Elsaid et al. (61), the developmental pathways for most tissue-resident cells do not fit the rudimentary model of adult hematopoiesis, but instead, follow a highly conserved and layered approach during ontogeny. These cells arise in distinct waves in coordination with tissue development, thus accounting for both the spatial and temporal aspects of embryogenesis. It is thought that highly coordinated programs and interactions between the stromal and immune cells facilitate tissue development, in addition to providing early immune protection specific to a given tissue type (61).

Considering the fact that BM ILC progenitors provide minimal contributions to tissue-resident ILC pools, it stands to reason that tissue-resident ILCs perhaps colonize during early stages in ontogeny rather than constantly being restored through BM lymphopoiesis. Intriguingly, the idea of layered ontogeny in ILC development has recently been explored in a lineage tracing study that closely monitored ILC2 development and turnover (47). Through elegant pulse-labeling of putative BM and fetal ILC precursors (Id2^+^ and Arg-1^+^ respectively) (30, 62), this group showed that development of ILC2s is temporally controlled and that they follow the model of layered lymphopoiesis similar to the one described in early macrophage development. These findings argue that ILC2s rapidly colonize peripheral tissues during the first week or two of postnatal life in mice and at a time when the bone marrow (BM) is still establishing itself as the sole source of hematopoietic progenitor activity. Strikingly, this study also concludes that once they establish residence in peripheral tissues, ILC2s turn over very slowly within the specific peripheral tissue microenvironments. This includes BM ILC2s, which appears to turnover with a kinetics of weeks to months rather than days. Thus, the evidence to suggest that BM ILC2s seed peripheral tissues to any large degree is increasingly scant and it is likely that the ILC2s present within the BM represent tissue-resident cells with tissue-specific function (Schneider et al., 2019). Indeed, a subsequent fate-mapping study using polychromic reporter mice expressing Id2, Bcl11b, GATA3, RORα and RORγt revealed co-differentiation of ILCs and developing thymocytes in embryonic thymi, strengthening the concept that ILCs develop early in life parallel to early T-cell development (36).

## TCR Gene Rearrangements in ILCs

V(D)J recombination at the TCR loci is an extraordinary process reserved for committed thymocytes at their DN stage. It is thought to occur in sequence as DN cells progress towards more committed DP state. TCR gene expression, as a unique hallmark of T-cell development, has hinted at a strikingly close relationship between ILCs and T cells in the past with both EILPs and ILCPs expressing high levels of sterile TCR transcripts even though they were originally identified in the adult BM (63). In order to concretely address whether ILCs stem from embryonic/neonatal T-cell development, we recently performed detailed genetic analyses of all TCR loci in mature tissue-resident ILCs. Single-cell analyses of cecal ILC1, 2 and 3s and lung ILC2s showed abundant expression of TCR constant region transcripts. Specifically, Cβ transcripts are expressed in all ILC subsets while Cα, γ and δ transcript levels are more closely associated with specific ILC subsets. Subsequent in-depth genomic analyses revealed that although lung ILC2s do not show evidence of DNA rearrangements at the TCRβ loci, they exhibit clear evidence of rearrangements at their TCRγ loci in a pattern that is strikingly similar to mature Vγ2^+^ γδ T cells. In addition, qPCR analysis shows that at least one of the TCRδ alleles is frequently deleted. Lastly, when sequenced, Vγ2-Jγ1 rearrangements were found to be largely out-of-frame, thus precluding their ability to express a functional TCRγ subunit even if this locus was actively transcribed and translated (64).

**Figure 2:**
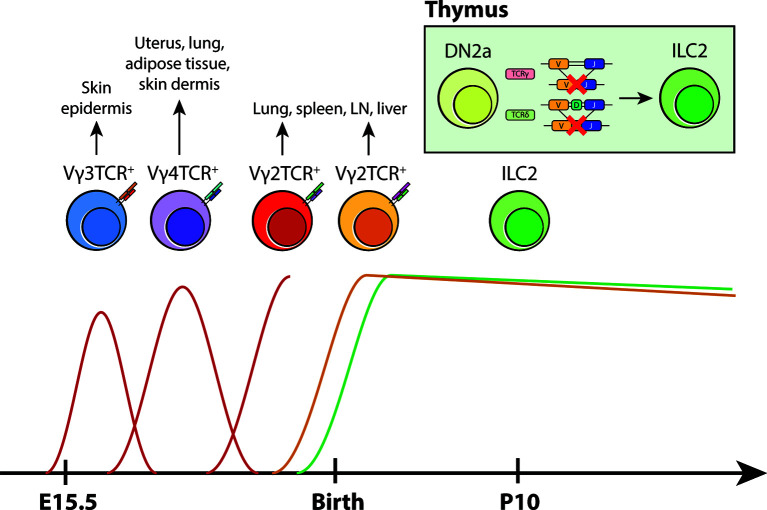
A new model for layered ontogeny of ILC2s along with fetal and postnatal waves of γδ T cells. Like most tissue-resident cells, the developmental timepoint in which ILC2s arise and colonize tissues coincides with γδ T cells early in life (based on layered ontogeny in B6 mice). Taken together with TCR locus sequence tracing data and pulse-labeling lineage tracing studies, it is highly likely that tissue-resident ILC2s arise perinatally from developing DN2s that have ineffectively rearranged their γ/δ loci, rather than from progenitors in the BM. These cells then take up residence at their designated tissue sites and self-renew locally.

The detailed characterization of TCR gene rearrangements performed in our study offers a fascinating window into the life history of ILC2s. The preferential expression of TCRγ constant regions and preponderance of non-productive TCRγ and δ gene rearrangements without the VDJ recombination of the TCRβ locus suggest the possibility that ILC2s may abortively arise from DN2-DN3 transition stage during γδ T-cell development. As reviewed in Spidale et al. (59), the development of tissue-resident γδ T cells occurs early in life in a time-sensitive manner. Unlike adult αβ T cells, γδ T cells emerge in progressive waves that are defined by the specific gene usage of Vγ and Vδ segments during fetal/neonatal development (58). Vγ3^+^ γδ T cells (also known as DETCs) are the first T cell subset to arise from the fetal liver during embryogenesis. They preferentially migrate to the epidermis and provide early immunity in developing skin. Subsequent γδ T cells that follow this vanguard wave of DETCs are Vγ4^+^ and Vγ2^+^ γδ T cells. Vγ4^+^ γδ T cells seed non-lymphoid tissue sites such as the uterus, lung, adipose tissue and skin dermis during late fetal development while Vγ2^+^ γδ T cells appear during the late fetal/neonatal stage, colonizing various mucosal and non-mucosal sites (59). In line with the layered model of ILC-genesis, it is possible that abortive Vγ2-Jγ1 locus rearrangements in tissue-resident ILC2s represent an ontogeneologic relic of “failed” Vγ2^+^ γδ T-cell development (**Figure 2**). When viewed in the context the aforementioned neonatal lineage tracing experiments (47) and the presence of ILCs in the embryonic thymus and their dependence on early T-cell transcription factors (36), a compelling case emerges for the development of ILCs from abortive T-cell development and as an offshoot from neonatal T-cell progenitors.

In addition to ILC2s, it is noteworthy that earlier studies showed the TCR loci are also frequently rearranged in NK cells. Comprehensive genomic analyses of adult and neonatal splenic NK cells revealed that the TCRγ locus is rearranged while TCRβ locus maintains its germline configuration. Intriguingly, unlike ILC2s, NK cells were reported to express rearranged TCRγ transcripts; however, the sequencing data exhibited a variable degree of productive rearrangements despite being isolated from mRNA products. Minimal TCRδ locus rearrangements (Vδ4-Jδ1) were detected and only in neonatal NK cells, suggesting that similar to ILC2s, at least one allele could have been deleted due to an abortive gene rearrangement event at the TCRα/δ loci (65).

Further evidence for TCR gene rearrangements in other ILC subsets has recently been demonstrated in human studies through sophisticated single-cell transcriptome analyses that revealed gene expression patterns associated with tissue-residency and migration in human ILCs (66). In this report, blood ILC1s (EOMES^-/+^) are shown to uphold the expression of T-cell related-genes such as CD3, CD4, CD5, CD6, CD27, LEF1. As such, the group investigated whether these cells and other ILC subsets express rearranged TCR transcripts, despite lacking surface TCRs. They showed that blood EOMES^+^ ILC1s express rearranged αβ chains and putative blood EOMES^-^ ILC1s exhibit rearrangements in all four TCR chains. Within this pool, ILC1s with rearranged δ/γ transcripts associated with *Ikzf2* expression while ILC1s with rearranged α/β transcripts clustered closely with CXCR3^+^ Th1 cells (66). These observations suggest that ILC1s are more akin to CD4^+^ and CD8^+^ T cells and that in their lifetime, they have undergone a maturation process in the thymus. Although it is noteworthy that, these authors failed to detect rearranged TCRγ chain expression in human ILC2s, it is also important to bear in mind that these studies only evaluated TCR *transcripts* rather than the genomic loci. Indeed, in our previous studies of murine ILC2 genomic loci and transcripts, we found that the rearranged loci are transcriptionally silent in murine ILC2s as well, potentially reflecting an attempt to silence alleles that have failed productive in-frame rearrangement; a process that naturally occurs in developing thymocytes. With this in mind, it would be of interest to now evaluate genomic TCR loci in human ILC subsets. Although human and murine ILC/T-cell biology exhibit significant differences, the use of TCR gene rearrangements as an indelible mark of the lineage of origin in both of these studies points to a thymic origin of the tissue-resident ILCs.

## Notch Signaling, the Initial Fate Determinator in the Thymus

**Figure 3:**
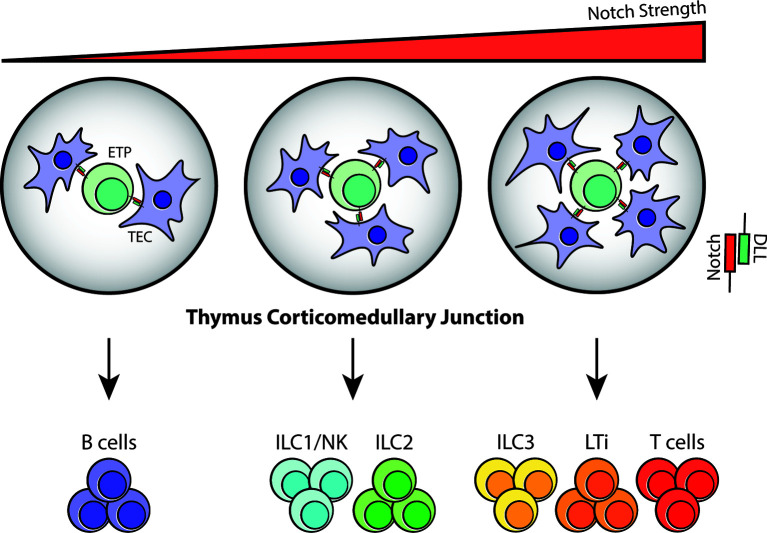
Notch signaling at the thymus corticomedullary junction may determine T-cell/ILC fate. Notch signaling is mediated through direct cell-cell contact in the thymus; therefore, the number of TECs interacting with TSPs/ETPs at the corticomedullary junction will vary depending on cell position and niche availability. Based on the observations from *in vitro* assays, it is highly likely that ETPs that receive zero to low Notch-DLL interaction will differentiate into B cells. In contrast, strong Notch signaling will guide ETPs towards the T-cell and ILC3/LTi lineages while intermediate Notch signaling will result in ILC1/NK and ILC2 commitment during fetal and postnatal periods.

In addition to abortive V(D)J recombination, limited Notch signaling in the thymic microenvironment may also determine whether thymocytes continue developing as T cells or shunt away from the T-cell lineage and into ILC lineages. Notch activity was discovered in the early 20^th^ century from strains of *Drosophila* that exhibited serrated “notched” wings (67). Its signaling pathway is evolutionarily conserved and is imperative for regulating cell fate decisions, survival, proliferation and niche formation (68). In the context of T-cell development, Notch1 and its ligand, Delta-like 4 (DLL4), serve as an essential checkpoint signal that imprints T-cell identity in TSPs entering the thymus. Their signaling is mediated by direct cell-cell interaction between TSPs and TECs and promotes the acquisition of T-cell fate by upregulating T-cell specific genes while gradually repressing myeloid and B-cell potential (49, 69). Upon interaction, a series of proteolytic cleavage events release the cytoplasmic domain of Notch, which then binds to recombination binding protein-J (RBP-J) in the nucleus and activates transcription of genes associated with T-cell development including *Tcf7*, *Hes1*, *Gata3* and *Bcl11b* (54, 69, 70). Loss-of-function studies of Notch1 and Cre-mediated deletion of DLL4 revealed aberrant proliferation of B cells in the thymus, and argues that their presence is indeed necessary for T-cell commitment (49). In contrast, the importance of Notch signaling in ILC development has been quite controversial. Deletion of RBP-Jκ in hematopoietic cells leads to a noticeable reduction in the frequency of lamina propria NKp46^+^ILC3, but not CD4^+^LTi-like cells (71, 72). Moreover, culturing EILPs on OP9-DLL1 stroma results in enhanced generation of ILC2s, yet the development of all ILC subsets is unaffected in the absence of Notch signaling (29). Lastly, multiple groups have concluded that committed ILC progenitors and precursors have diminished dependency on Notch (29, 31, 73), thus creating the notion that its signaling is dispensable in ILC development. More recently however, an *in vitro* assay performed using a Tet-inducible cell system, which allows for fine-tuning of DLL1 and DLL4 expression under doxycyclin, revealed that the strength and duration of Notch signaling influences the development of different ILC subsets. In this study, CLPs that were exposed to robust Notch-DLL signaling differentiated predominantly into T cells, LTi cells and ILC3s, whereas those receiving intermediate signaling preferentially became ILC1s/NK cells and ILC2s. Expectedly, CLPs with minimal to no Notch signaling led to B-cell differentiation (**Figure 3**). The same study also showed that specific deletion of DLL4 on TECs results in an abnormal expansion of ILC2 in the thymus (38), suggesting that the gradient of Notch signaling may influence ILC fate in the thymic microenvironment.

Notch signaling is not binary, but rather it is dose- and time-dependent during ontogeny and in cell fate decisions (68). It is widely known that TSPs/ETPs are multipotent, and that T-cell potential is acquired progressively through DN to DP stage. Considering the fact that Notch signaling is facilitated by cell-cell interaction, it is highly likely that the size of initial thymic niche for ETPs is limited. Therefore, the degree and duration of Notch signaling would vary from cell to cell, leaving the option for other lineages to appear in the embryonic/postnatal thymus similar to the ones observed in the dose-dependent *in vitro* system (**Figure 3**). Addressing the redundancy of Notch in later ILC progenitors requires careful consideration of its unique purpose. Notch and its ligand interaction are often described in the context of development and regulation of cell fates. In both T-cell and ILC development, Notch establishes T-cell and ILC identity through upregulation of TCF-1 and its related transcription factors. As DN2 cells mature into DN3 cells, their dependency on Notch signaling decreases, eventually becoming redundant (74). Since a similar behavior is observed in downstream ILC progenitors, it is possible that TCF-1^+^ EILPs and CHILPs are already committed and downstream of developing thymocytes that share the same hierarchical level as DN3 cells in the differentiation hierarchy.

## E-Id Proteins and Bcl11b Determine Thymocyte Fate

**Figure 4:**
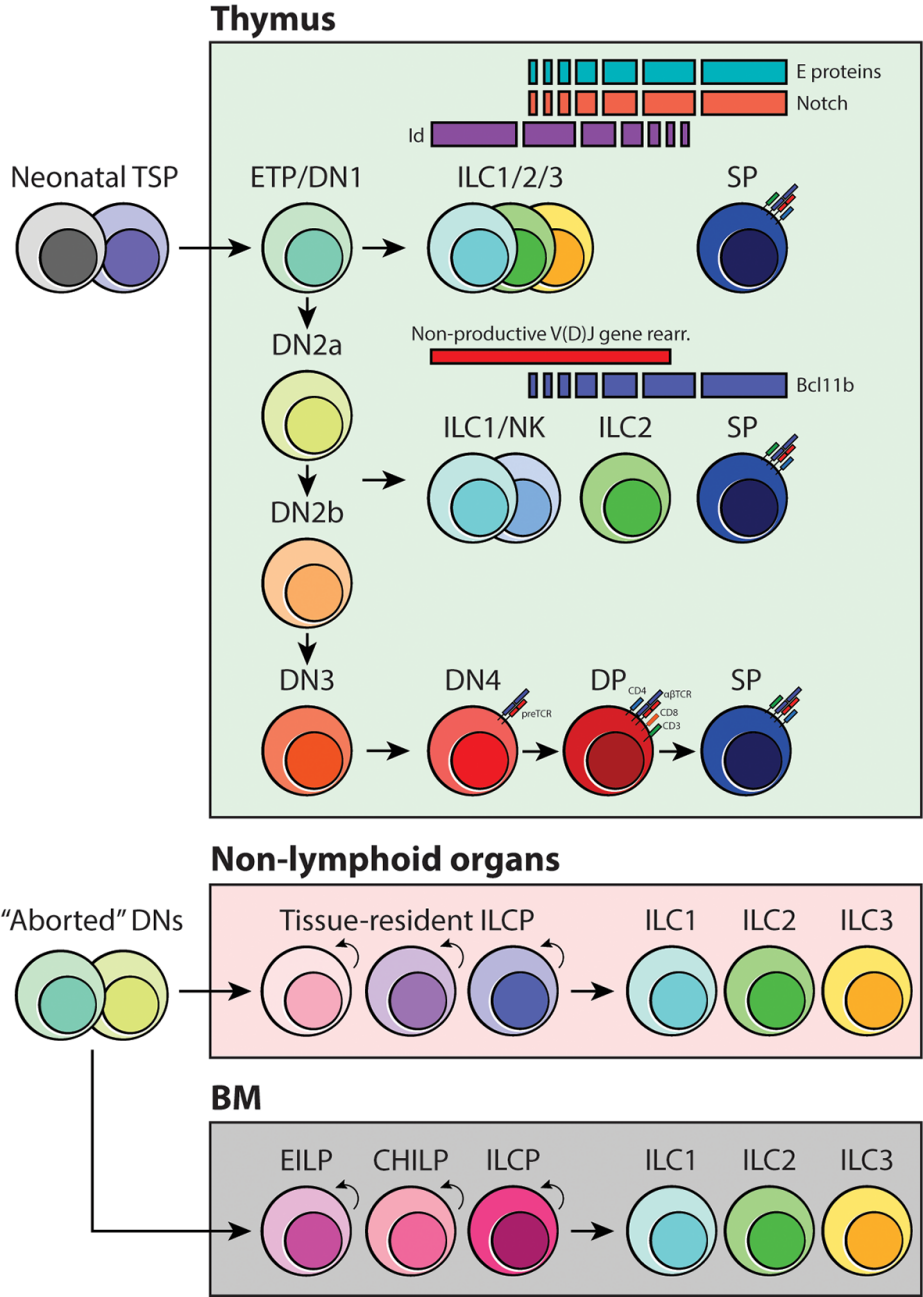
Schematic of ILC development in coordination with thymocyte specification and maturation. As early T-cell progenitors are known to be multipotent, we propose that the branchpoint in which ILC differentiation occurs is at the DN1/ETP and DN2-DN3 transition stage. Depending on the status of the TCR loci, strength of Notch signaling and activities of E-Id proteins and Bcl11b, developing thymocytes may acquire innate-like properties and give rise to one of three ILC subsets. Furthermore, we suggest that tissue-resident ILC progenitors, including the BM, originate from failed T-cell development and locally maintain the mature ILC pool.

The transcription factors involved in positive and negative regulation of ILC differentiation have been discussed in great detail in other reviews (19, 69, 75). Emerging studies have highlighted the fact that differential expression of factors associated with T-cell development, HES1, TCF1 and GATA3, RORα, E and Id proteins, and Bcl11b, are all critical for ILC maturation (19, 36, 76–78). Among these, E-Id proteins and Bcl11b are especially interesting because they have been shown to play a role in inducing ILC development in the thymus. E proteins are under the class I basic helix-loop-helix (bHLH) family of transcription factors that are historically described in early T-cell lineage commitment and specification in coordination with Notch signaling. They form homo- or heterodimers (e.g., E2A-HEB) with other bHLH or HLH proteins, which then turns on the T-cell-specific program in developing thymocytes. E protein function is regulated by class IV HLH family inhibitor of DNA binding (Id) factors. Id proteins lack the DNA binding domain; thus, they sequester E protein function by forming a heterodimer complex that cannot bind to DNA (69). Intriguingly, hindering the activity of E proteins by deleting E2A and HEB or ectopically expressing Id1 or Id2 blocks the generation of functional T cells, and instead promotes ILC2 proliferation in adult thymi (76, 79, 80). Furthermore, fetal ETPs lacking E2A and HEB *in vitro* can also generate ILC1- and ILC3-like cells (76); therefore, it is highly likely that the strength of Notch signaling, and E-Id protein levels may influence ETP fate in the thymus (**Figure 4**). Bcl11b, on the other hand, belongs to a family of Kruppel-like C_2_H_2_ type zinc finger transcription factors and is expressed in late DN2a thymocytes and essential for DN2-DN3 transition in T-cell development (69, 81). *Bcl11b*-deficiency causes arrest of T cells at the DN2a stage and, fascinatingly, allows aberrant differentiation of NKp46^+^ ILC1/NK cells and myeloid cells in the thymus (82, 83). Bcl11b has also been implicated ILC2 biology and development. It is widely accepted that Bcl11b is important for overall ILC2 maintenance and function; however, in the original study that used heterozygous germline knockout and conditional knockout of *Bcl11b*, only the ST2^+^ BM ILC2 compartment was reduced upon *Bcl11b* deletion. Surprisingly, the frequency of lung-resident ST2^+^ ILC2s was not affected by this genetic manipulation, but rather there was an incredible increase in the KLRG1^+^ ILC2 population (84). It would be extremely interesting to revisit these transgenic mice models and examine their thymus-residing cells to determine if there is an abnormal expansion of KLRG1^+^ ILC2s or perhaps other ILC subsets in the thymus through high fidelity single-cell sequencing. Considering this and the evidence for layered ontogeny of ILCs, it is quite possible that the origin of BM ILC2s and lung ILC2s is completely independent of each other and that E-Id proteins and Bcl11b may be the transcriptional checkpoints that initiate reprogramming of T cells that have neither received adequate Notch signaling nor undergone productive TCR gene rearrangements (**Figure 4**).

## Addressing Normal Development of ILCs in T-Cell Knockout Models

The presence of functional ILC subsets in mice with genetic lesions in T-cell development raises an interesting conundrum because if ILCs are indeed associated with T-cell development, one might predict there would be a reduction in their frequency upon interfering with thymocyte maturation. In contrast, earlier characterization of ILCs has indicated that ILCs can develop and function normally in *Rag* ^-/-^ and nude mice, thus highlighting them as distinct subsets, independent of the T-cell lineage (85). With the evidence of non-productive TCR rearrangement, however, it is now critical to revisit this hypothesis and re-evaluate it from the perspective of abortive T-cell development. In maturing thymocytes, RAG genes are typically expressed during the DN2-DN3 transition (49) (**Figure 1**). Therefore, without active RAG1 or RAG2 proteins, thymocytes cannot rearrange their TCR loci and progress further into latter stages of T-cell development (86–88). Taken together with the fact that pre-DN3 cells are multipotent, there is room for *Rag*-deficient ETPs and DN2a cells to shunt away from the T-cell lineage and immediately choose the innate cell fate as part of a salvage mechanism. Indeed, our characterization of ILC2s in *Rag1^-/-^* mice revealed that there is a heightened frequency of ILC2s in the thymus, indicating active generation of ILC2s upon blocking TCR gene rearrangements. Likewise, other studies have also characterized the heterogeneous populations of DN cells in RAG mutants containing putative NK cells (89), and suggesting that this pathway may apply to other ILC subsets. Similar to RAG mutants, the mature T-cell population is also substantially decreased in nude mice carrying null mutations of the forkhead transcription factor (FOXN1), which is essential for TEC differentiation during development (90). The nude mouse vestigial thymic microenvironment is detrimental for early T-cell progenitors as it cannot provide sufficient Notch signaling for their specification and survival. Considering the evidence of dose-dependent Notch signaling in determining cell fate, it is highly likely that all T-cell progenitors are co-opted into the ILC fate program immediately after entering this non-functional thymic niche. This would increase the total frequency of ILCs in the peripheral tissues, and certainly, previous studies have observed heighten ILC counts at various barrier sites (91, 92). In future studies, it would be extremely interesting to purposefully disrupt TCR gene rearrangement at specific stages in development and investigate whether this influences the total number of ILCs in the periphery.

## Functional Significance of BM ILCs

The vast majority of ILCs within the BM are affiliated with the ILC2 subset. Given the evidence that ILCs can develop neonatally from thymic precursors and become tissue-resident thereafter, the functional significance of BM ILC2s then comes into question. Likewise, the fact that these cells show a remarkably slow turnover (47) would argue against them serving as a precursor pool for peripheral ILCs and might instead suggest that, like peripheral ILCs, these cells fulfill an important tissue-resident function. Consistent with this observation, it has been shown that self-renewal and maintenance of ILCs is facilitated by a pre-existing pool of tissue-resident ILCPs under inflammatory conditions (45, 48). Specifically, during the acute phase of *Nippostrongylus brasiliensis* helminth infection, ILC2s in the lung, gut and mesenteric lymph node proliferate locally without significant contribution from the BM (45, 47, 93). The signs of recruitment and redistribution of ILC2s from other tissues including the BM only begin to appear after day 15 post-infection where the acute inflammatory conditions turn chronic and overt (45, 93, 94). Recent studies have shown that their contribution is rather minor (<10%) and that immature *Il18r1* expressing BM ILCPs are responsible for seeding BM-derived ILC2s in the lung to generate the full phenotypic spectrum of ILC2s (47, 93). Indeed, there is a clear precedent for a selective BM-resident function for several mature hematopoietic lineages. Macrophage-like osteoclasts, for example, play a key role in bone remodeling and, together with osteoblasts regulate bone homeostasis (95). Similarly, the BM serves as a long-term reservoir and archive for antigen-specific, antibody-secreting plasma cells and possibly a unique population of isotype-switched, affinity-mature memory B cells that could be called into service upon reinfection with specific pathogens (96, 97). With these examples in mind, it is worth considering a BM specific role for resident ILC2s. Intriguingly, several studies have suggested that BM ILC2s can, in fact, play key roles in stimulating eosinophilopoiesis in response to system Th2 inflammatory insults (98, 99). Likewise, recent studies suggest that through secretion of GM-CSF ILC2s can stimulate the recovery of BM hematopoiesis in response severe chemically induced stress (100). In aggregate, these studies suggest that, like there peripheral tissue counterparts, BM ILC2s may have colonized this tissue early in development and serve a tissue-resident purpose thereafter. Taken together with previous TCR gene rearrangement data, it will now be important to test whether these cells, too, show genetic marks of deviation from early thymic progenitors.

## Concluding Remarks

The recent discovery of ILCs has been transformative in our understanding of the development of finally orchestrated and appropriate immune responses to the appropriate pathogens and in bridging the division of labor between innate and adaptive immune responses. More recently, their functional significance has been expanded to include roles in non-barrier organs and key roles in tissue and organ homeostasis and repair. Despite this attention and these insights, a deep understanding of their ontogeny and development has lagged behind. Accumulating evidence now suggests remarkable conservation of molecular, transcriptional, and developmental parallels between these cells and neonatal T cells and, indeed, that in some instances these cells can develop from T cells that have failed to appropriately rearrange their antigen specific receptors. Studies of layered ontogeny of T cells and ILCs point towards the fact that long-lived, tissue-resident ILCs are likely to be thymus-derived and that during steady-state or mild immune challenge, they expand and respond appropriately to remaining inflammation. However, in hematopoietic crisis, for example chronic inflammation, complete hematopoietic ablation, sepsis, or severe viral infections, BM stem cell derived ILCs may also be called into service and enter the peripheral niches to support the existing pool of tissue-resident ILC subsets. Certainly, future studies are needed to further clarify the relative contributions of these pools, their lineage relationships and whether they can be harnessed for improved treatment of clinical disease.

## Author Contributions

SBS and KMM wrote the manuscript and SBS designed the figures. All authors contributed to the article and approved the submitted version.

## Funding

This work was funded by grant numbers PJT-148681 and PJT-156235 from the Canadian Institutes of Health Research (CIHR). SBS was supported by an AllerGen Network Centre of Excellence and CIHR Frederick Banting & Charles Best Canada Graduate Scholarship–Master’s Program (CGS-M) Scholarship.

## Conflict of Interest

The authors declare that the research was conducted in the absence of any commercial or financial relationships that could be construed as a potential conflict of interest.

## References

[1] Kiessling R Klein E Pross H Wigzell H. “Natural” Killer Cells in the Mouse. II. Cytotoxic Cells With Specificity for Mouse Moloney Leukemia Cells. Characteristics of the Killer Cell. Eur J Immunol (1975) 5(2):117–21. 10.1002/eji.1830050209 1086218

[2] Mebius RE Rennert P Weissman IL. Developing Lymph Nodes Collect CD4+CD3- Ltbeta+ Cells That can Differentiate to APC, NK Cells, and Follicular Cells But Not T or B Cells. Immunity (1997) 7(4):493–504. 10.1016/s1074-7613(00)80371-4 9354470

[3] Spits H Di Santo JP. The Expanding Family of Innate Lymphoid Cells: Regulators and Effectors of Immunity and Tissue Remodeling. Nat Immunol (2011) 12(1):21–7. 10.1038/ni.1962 21113163

[4] Sonnenberg GF Artis D. Innate Lymphoid Cell Interactions With Microbiota: Implications for Intestinal Health and Disease. Immunity (2012) 37(4):601–10. 10.1016/j.immuni.2012.10.003 PMC3495160 23084357

[5] Robinette ML Colonna M. Innate Lymphoid Cells and the MHC. HLA (2016) 87(1):5–11. 10.1111/tan.12723 26812060 PMC5658205

[6] Vivier E van de Pavert SA Cooper MD Belz GT. The Evolution of Innate Lymphoid Cells. Nat Immunol (2016) 17(7):790–4. 10.1038/ni.3459 PMC5287353 27328009

[7] Mohammadi H Sharafkandi N Hemmatzadeh M Azizi G Karimi M Jadidi-Niaragh F. The Role of Innate Lymphoid Cells in Health and Disease. J Cell Physiol (2018) 233(6):4512–29. 10.1002/jcp.26250 29058773

[8] Licona-Limon P Kim LK Palm NW Flavell RA. TH2, Allergy and Group 2 Innate Lymphoid Cells. Nat Immunol (2013) 14(6):536–42. 10.1038/ni.2617 23685824

[9] Gold MJ Antignano F Halim TY Hirota JA Blanchet MR Zaph C. Group 2 Innate Lymphoid Cells Facilitate Sensitization to Local, But Not Systemic, TH2-inducing Allergen Exposures. J Allergy Clin Immunol (2014) 133(4):1142–8. 10.1016/j.jaci.2014.02.033 24679471

[10] Lo BC Gold MJ Hughes MR Antignano F Valdez Y Zaph C. The Orphan Nuclear Receptor RORalpha and Group 3 Innate Lymphoid Cells Drive Fibrosis in a Mouse Model of Crohn’s Disease. Sci Immunol (2016) 1(3):eaaf8864. 10.1126/sciimmunol.aaf8864 PMC5489332 28670633

[11] Ebbo M Crinier A Vely F Vivier E. Innate Lymphoid Cells: Major Players in Inflammatory Diseases. Nat Rev Immunol (2017) 17(11):665–78. 10.1038/nri.2017.86 28804130

[12] Halim TY Krauss RH Sun AC Takei F. Lung Natural Helper Cells are a Critical Source of Th2 Cell-Type Cytokines in Protease Allergen-Induced Airway Inflammation. Immunity (2012) 36(3):451–63. 10.1016/j.immuni.2011.12.020 22425247

[13] Bruchard M Ghiringhelli F. Deciphering the Roles of Innate Lymphoid Cells in Cancer. Front Immunol (2019) 10:656. 10.3389/fimmu.2019.00656 31024531 PMC6462996

[14] Wang H Shen L Sun X Liu F Feng W Jiang C. Adipose Group 1 Innate Lymphoid Cells Promote Adipose Tissue Fibrosis and Diabetes in Obesity. Nat Commun (2019) 10(1):3254. 10.1038/s41467-019-11270-1 31332184 PMC6646407

[15] Withers DR. Innate Lymphoid Cell Regulation of Adaptive Immunity. Immunology (2016) 149(2):123–30. 10.1111/imm.12639 PMC5011676 27341319

[16] Messing M Jan-Abu SC McNagny K. Group 2 Innate Lymphoid Cells: Central Players in a Recurring Theme of Repair and Regeneration. Int J Mol Sci (2020) 21(4):1350. 10.3390/ijms21041350 PMC7072936 32079296

[17] Panda SK Colonna M. Innate Lymphoid Cells in Mucosal Immunity. Front Immunol (2019) 10:861. 10.3389/fimmu.2019.00861 31134050 PMC6515929

[18] Cherrier DE Serafini N Di Santo JP. Innate Lymphoid Cell Development: A T Cell Perspective. Immunity (2018) 48(6):1091–103. 10.1016/j.immuni.2018.05.010 29924975

[19] Diefenbach A Colonna M Koyasu S. Development, Differentiation, and Diversity of Innate Lymphoid Cells. Immunity (2014) 41(3):354–65. 10.1016/j.immuni.2014.09.005 PMC4171710 25238093

[20] Fuchs A. ILC1s in Tissue Inflammation and Infection. Front Immunol (2016) 7:104. 10.3389/fimmu.2016.00104 27047491 PMC4801855

[21] Fuchs A Vermi W Lee JS Lonardi S Gilfillan S Newberry RD. Intraepithelial Type 1 Innate Lymphoid Cells are a Unique Subset of IL-12- and IL-15-responsive IFN-Gamma-Producing Cells. Immunity (2013) 38(4):769–81. 10.1016/j.immuni.2013.02.010 PMC3634355 23453631

[22] Monticelli LA Sonnenberg GF Abt MC Alenghat T Ziegler CG Doering TA. Innate Lymphoid Cells Promote Lung-Tissue Homeostasis After Infection With Influenza Virus. Nat Immunol (2011) 12(11):1045–54. 10.1031/ni.2131 PMC3320042 21946417

[23] Wilhelm C Hirota K Stieglitz B Van Snick J Tolaini M Lahl K. An IL-9 Fate Reporter Demonstrates the Induction of an Innate IL-9 Response in Lung Inflammation. Nat Immunol (2011) 12(11):1071–7. 10.1038/ni.2133 PMC3198843 21983833

[24] Lo BC Canals Hernaez D Scott RW Hughes MR Shin SB Underhill TM. The Transcription Factor RORalpha Preserves ILC3 Lineage Identity and Function During Chronic Intestinal Infection. J Immunol (2019) 203(12):3209–15. 10.4049/jimmunol.1900781 31676672

[25] Zhou W Sonnenberg GF. Activation and Suppression of Group 3 Innate Lymphoid Cells in the Gut. Trends Immunol (2020) 41(8):721–33. 10.1016/j.it.2020.06.009 PMC7395873 32646594

[26] Cherrier M Eberl G. The Development of LTi Cells. Curr Opin Immunol (2012) 24(2):178–83. 10.1016/j.coi.2012.02.003 22386930

[27] Halim TY Steer CA Matha L Gold MJ Martinez-Gonzalez I McNagny KM. Group 2 Innate Lymphoid Cells are Critical for the Initiation of Adaptive T Helper 2 Cell-Mediated Allergic Lung Inflammation. Immunity (2014) 40(3):425–35. 10.1016/j.immuni.2014.01.011 PMC4210641 24613091

[28] Moro K Yamada T Tanabe M Takeuchi T Ikawa T Kawamoto H. Innate Production of T(H)2 Cytokines by Adipose Tissue-Associated c-Kit(+)Sca-1(+) Lymphoid Cells. Nature (2010) 463(7280):540–4. 10.1038/nature08636 20023630

[29] Yang Q Li F Harly C Xing S Ye L Xia X. TCF-1 Upregulation Identifies Early Innate Lymphoid Progenitors in the Bone Marrow. Nat Immunol (2015) 16(10):1044–50. 10.1038/ni.3248 PMC4575643 26280998

[30] Klose CSN Flach M Mohle L Rogell L Hoyler T Ebert K. Differentiation of Type 1 ILCs From a Common Progenitor to All Helper-Like Innate Lymphoid Cell Lineages. Cell (2014) 157(2):340–56. 10.1016/j.cell.2014.03.030 24725403

[31] Possot C Schmutz S Chea S Boucontet L Louise A Cumano A. Notch Signaling is Necessary for Adult, But Not Fetal, Development of RORgammat(+) Innate Lymphoid Cells. Nat Immunol (2011) 12(10):949–58. 10.1038/ni.2105 21909092

[32] Yu X Wang Y Deng M Li Y Ruhn KA Zhang CC. The Basic Leucine Zipper Transcription Factor NFIL3 Directs the Development of a Common Innate Lymphoid Cell Precursor. Elife (2014) 3:e04406. 10.7554/eLife.04406 PMC4356142 25310240

[33] Xu W Domingues RG Fonseca-Pereira D Ferreira M Ribeiro H Lopez-Lastra S. NFIL3 Orchestrates the Emergence of Common Helper Innate Lymphoid Cell Precursors. Cell Rep (2015) 10(12):2043–54. 10.1016/j.celrep.2015.02.057 25801035

[34] Seillet C Rankin LC Groom JR Mielke LA Tellier J Chopin M. Nfil3 is Required for the Development of All Innate Lymphoid Cell Subsets. J Exp Med (2014) 211(9):1733–40. 10.1084/jem.20140145 PMC4144736 25092873

[35] Jones R Cosway EJ Willis C White AJ Jenkinson WE Fehling HJ. Dynamic Changes in Intrathymic ILC Populations During Murine Neonatal Development. Eur J Immunol (2018) 48(9):1481–91. 10.1002/eji.201847511 PMC6174991 29851080

[36] Ferreira ACF Szeto ACH Heycock MWD Clark PA Walker JA Crisp A. Roralpha is a Critical Checkpoint for T Cell and ILC2 Commitment in the Embryonic Thymus. Nat Immunol (2021) 22(2):166–78. 10.1038/s41590-020-00833-w PMC7116838 33432227

[37] Yang Q Bhandoola A. The Development of Adult Innate Lymphoid Cells. Curr Opin Immunol (2016) 39:114–20. 10.1016/j.coi.2016.01.006 PMC4801723 26871595

[38] Koga S Hozumi K Hirano KI Yazawa M Terooatea T Minoda A. Peripheral PDGFRalpha(+)gp38(+) Mesenchymal Cells Support the Differentiation of Fetal Liver-Derived ILC2. J Exp Med (2018) 215(6):1609–26. 10.1084/jem.20172310 PMC5987924 29728440

[39] Cumano A Berthault C Ramond C Petit M Golub R Bandeira A. New Molecular Insights Into Immune Cell Development. Annu Rev Immunol (2019) 37:497–519. 10.1146/annurev-immunol-042718-041319 31026413

[40] Chea S Schmutz S Berthault C Perchet T Petit M Burlen-Defranoux O. Single-Cell Gene Expression Analyses Reveal Heterogeneous Responsiveness of Fetal Innate Lymphoid Progenitors to Notch Signaling. Cell Rep (2016) 14(6):1500–16. 10.1016/j.celrep.2016.01.015 26832410

[41] Bando JK Liang HE Locksley RM. Identification and Distribution of Developing Innate Lymphoid Cells in the Fetal Mouse Intestine. Nat Immunol (2015) 16(2):153–60. 10.1038/ni.3057 PMC4297560 25501629

[42] Sawa S Lochner M Satoh-Takayama N Dulauroy S Berard M Kleinschek M. Rorgammat+ Innate Lymphoid Cells Regulate Intestinal Homeostasis by Integrating Negative Signals From the Symbiotic Microbiota. Nat Immunol (2011) 12(4):320–6. 10.1038/ni.2002 21336274

[43] Tang Y Peitzsch C Charoudeh HN Cheng M Chaves P Jacobsen SE. Emergence of NK-cell Progenitors and Functionally Competent NK-cell Lineage Subsets in the Early Mouse Embryo. Blood (2012) 120(1):63–75. 10.1182/blood-2011-02-337980 22072559

[44] Bai L Vienne M Tang L Kerdiles Y Etiennot M Escaliere B. Liver Type 1 Innate Lymphoid Cells Develop Locally Via an Interferon-Gamma-Dependent Loop. Science (2021) 371(6536):eaba4177. 10.1126/science.aba4177 33766856

[45] Gasteiger G Fan X Dikiy S Lee SY Rudensky AY. Tissue Residency of Innate Lymphoid Cells in Lymphoid and Nonlymphoid Organs. Science (2015) 350(6263):981–5. 10.1126/science.aac9593 PMC4720139 26472762

[46] Moro K Kabata H Tanabe M Koga S Takeno N Mochizuki M. Interferon and IL-27 Antagonize the Function of Group 2 Innate Lymphoid Cells and Type 2 Innate Immune Responses. Nat Immunol (2016) 17(1):76–86. 10.1038/ni.3309 26595888

[47] Schneider C Lee J Koga S Ricardo-Gonzalez RR Nussbaum JC Smith LK. Tissue-Resident Group 2 Innate Lymphoid Cells Differentiate by Layered Ontogeny and In Situ Perinatal Priming. Immunity (2019) 50(6):1425–38.e5. 10.1016/j.immuni.2019.04.019 31128962 PMC6645687

[48] Ghaedi M Shen ZY Orangi M Martinez-Gonzalez I Wei L Lu X. Single-Cell Analysis of RORalpha Tracer Mouse Lung Reveals ILC Progenitors and Effector ILC2 Subsets. J Exp Med (2020) 217(3):e20182293. 10.1084/jem.20182293 PMC7062532 31816636

[49] Koch U Radtke F. Mechanisms of T Cell Development and Transformation. Annu Rev Cell Dev Biol (2011) 27:539–62. 10.1146/annurev-cellbio-092910-154008 21740230

[50] Adolfsson J Mansson R Buza-Vidas N Hultquist A Liuba K Jensen CT. Identification of Flt3+ Lympho-Myeloid Stem Cells Lacking Erythro-Megakaryocytic Potential a Revised Road Map for Adult Blood Lineage Commitment. Cell (2005) 121(2):295–306. 10.1016/j.cell.2005.02.013 15851035

[51] Lai AY Kondo M. Asymmetrical Lymphoid and Myeloid Lineage Commitment in Multipotent Hematopoietic Progenitors. J Exp Med (2006) 203(8):1867–73. 10.1084/jem.20060697 PMC2118384 16880261

[52] Bell JJ Bhandoola A. The Earliest Thymic Progenitors for T Cells Possess Myeloid Lineage Potential. Nature (2008) 452(7188):764–7. 10.1038/nature06840 18401411

[53] Ciofani M Zuniga-Pflucker JC. Determining Gammadelta Versus Alphass T Cell Development. Nat Rev Immunol (2010) 10(9):657–63. 10.1038/nri2820 20725107

[54] Shah DK Zuniga-Pflucker JC. An Overview of the Intrathymic Intricacies of T Cell Development. J Immunol (2014) 192(9):4017–23. 10.4049/jimmunol.1302259 24748636

[55] Rothenberg EV Moore JE Yui MA. Launching the T-cell-lineage Developmental Programme. Nat Rev Immunol (2008) 8(1):9–21. 10.1038/nri2232 18097446 PMC3131407

[56] Bresnick EH Hewitt KJ Mehta C Keles S Paulson RF Johnson KD. Mechanisms of Erythrocyte Development and Regeneration: Implications for Regenerative Medicine and Beyond. Development (2018) 145(1):dev151423. 10.1242/dev.151423 PMC5825862 29321181

[57] Hashimoto D Chow A Noizat C Teo P Beasley MB Leboeuf M. Tissue-Resident Macrophages Self-Maintain Locally Throughout Adult Life With Minimal Contribution From Circulating Monocytes. Immunity (2013) 38(4):792–804. 10.1016/j.immuni.2013.04.004 23601688 PMC3853406

[58] Xiong N Raulet DH. Development and Selection of Gammadelta T Cells. Immunol Rev (2007) 215:15–31. 10.1111/j.1600-065X.2006.00478.x 17291276

[59] Spidale NA Frascoli M Kang J. gammadeltaTCR-independent Origin of Neonatal Gammadelta T Cells Prewired for IL-17 Production. Curr Opin Immunol (2019) 58:60–7. 10.1016/j.coi.2019.04.011 PMC7147991 31128446

[60] Li Z Liu S Xu J Zhang X Han D Liu J. Adult Connective Tissue-Resident Mast Cells Originate From Late Erythro-Myeloid Progenitors. Immunity (2018) 49(4):640–53.e5. 10.1016/j.immuni.2018.09.023 30332630

[61] Elsaid R Soares-da-Silva F Peixoto M Amiri D Mackowski N Pereira P. Hematopoiesis: A Layered Organization Across Chordate Species. Front Cell Dev Biol (2020) 8:606642. 10.3389/fcell.2020.606642 33392196 PMC7772317

[62] Constantinides MG McDonald BD Verhoef PA Bendelac A. A Committed Precursor to Innate Lymphoid Cells. Nature (2014) 508(7496):397–401. 10.1038/nature13047 24509713 PMC4003507

[63] Harly C Cam M Kaye J Bhandoola A. Development and Differentiation of Early Innate Lymphoid Progenitors. J Exp Med (2018) 215(1):249–62. 10.1084/jem.20170832 PMC5748853 29183988

[64] Shin SB Lo BC Ghaedi M Scott RW Li Y Messing M. Abortive gammadeltaTCR Rearrangements Suggest ILC2s are Derived From T-cell Precursors. Blood Adv (2020) 4(21):5362–72. 10.1182/bloodadvances.2020002758 PMC7656916 33137203

[65] Veinotte LL Greenwood CP Mohammadi N Parachoniak CA Takei F. Expression of Rearranged TCRgamma Genes in Natural Killer Cells Suggests a Minor Thymus-Dependent Pathway of Lineage Commitment. Blood (2006) 107(7):2673–9. 10.1182/blood-2005-07-2797 16317098

[66] Mazzurana L Czarnewski P Jonsson V Wigge L Ringner M Williams TC. Tissue-Specific Transcriptional Imprinting and Heterogeneity in Human Innate Lymphoid Cells Revealed by Full-Length Single-Cell RNA-Sequencing. Cell Res (2021). 10.1038/s41422-020-00445-x PMC8089104 33420427

[67] Morgan TH. The Theory of the Gene. etc., New Haven: Yale University Press (1926).

[68] Maillard I Fang T Pear WS. Regulation of Lymphoid Development, Differentiation, and Function by the Notch Pathway. Annu Rev Immunol (2005) 23:945–74. 10.1146/annurev.immunol.23.021704.115747 15771590

[69] Hosokawa H Rothenberg EV. How Transcription Factors Drive Choice of the T Cell Fate. Nat Rev Immunol (2021) 21(3):162–76. 10.1038/s41577-020-00426-6 PMC7933071 32918063

[70] Radtke F Wilson A MacDonald HR. Notch Signaling in T- and B-cell Development. Curr Opin Immunol (2004) 16(2):174–9. 10.1016/j.coi.2004.01.002 15023410

[71] Georgiades P Ogilvy S Duval H Licence DR Charnock-Jones DS Smith SK. VavCre Transgenic Mice: A Tool for Mutagenesis in Hematopoietic and Endothelial Lineages. Genesis (2002) 34(4):251–6. 10.1002/gene.10161 12434335

[72] Lee JS Cella M McDonald KG Garlanda C Kennedy GD Nukaya M. AHR Drives the Development of Gut ILC22 Cells and Postnatal Lymphoid Tissues Via Pathways Dependent on and Independent of Notch. Nat Immunol (2011) 13(2):144–51. 10.1038/ni.2187 PMC3468413 22101730

[73] Seehus CR Aliahmad P de la Torre B Iliev ID Spurka L Funari VA. The Development of Innate Lymphoid Cells Requires TOX-dependent Generation of a Common Innate Lymphoid Cell Progenitor. Nat Immunol (2015) 16(6):599–608. 10.1038/ni.3168 25915732 PMC4439271

[74] Rothenberg EV Ungerback J Champhekar A. Forging T-Lymphocyte Identity: Intersecting Networks of Transcriptional Control. Adv Immunol (2016) 129:109–74. 10.1016/bs.ai.2015.09.002 PMC4747653 26791859

[75] Rothenberg EV. Single-Cell Insights Into the Hematopoietic Generation of T-lymphocyte Precursors in Mouse and Human. Exp Hematol (2021) 95:1–12. 10.1016/j.exphem.2020.12.005 33454362 PMC8018899

[76] Miyazaki M Miyazaki K Chen K Jin Y Turner J Moore AJ. The E-Id Protein Axis Specifies Adaptive Lymphoid Cell Identity and Suppresses Thymic Innate Lymphoid Cell Development. Immunity (2017) 46(5):818–34.e4. 10.1016/j.immuni.2017.04.022 28514688 PMC5512722

[77] Califano D Cho JJ Uddin MN Lorentsen KJ Yang Q Bhandoola A. Transcription Factor Bcl11b Controls Identity and Function of Mature Type 2 Innate Lymphoid Cells. Immunity (2015) 43(2):354–68. 10.1016/j.immuni.2015.07.005 PMC4657441 26231117

[78] Hosokawa H Romero-Wolf M Yang Q Motomura Y Levanon D Groner Y. Cell Type-Specific Actions of Bcl11b in Early T-lineage and Group 2 Innate Lymphoid Cells. J Exp Med (2020) 217(1):e20190972. 10.1084/jem.20190972 PMC7037248 31653691

[79] Wang HC Qian L Zhao Y Mengarelli J Adrianto I Montgomery CG. Downregulation of E Protein Activity Augments an ILC2 Differentiation Program in the Thymus. J Immunol (2017) 198(8):3149–56. 10.4049/jimmunol.1602009 PMC5404348 28258196

[80] Qian L Bajana S Georgescu C Peng V Wang HC Adrianto I. Suppression of ILC2 Differentiation From Committed T Cell Precursors by E Protein Transcription Factors. J Exp Med (2019) 216(4):884–99. 10.1084/jem.20182100 PMC6446881 30898894

[81] Kominami R. Role of the Transcription Factor Bcl11b in Development and Lymphomagenesis. Proc Jpn Acad Ser B Phys Biol Sci (2012) 88(3):72–87. 10.2183/pjab.88.72 PMC3365246 22450536

[82] Li P Burke S Wang J Chen X Ortiz M Lee SC. Reprogramming of T Cells to Natural Killer-Like Cells Upon Bcl11b Deletion. Science (2010) 329(5987):85–9. 10.1126/science.1188063 PMC3628452 20538915

[83] Li L Leid M Rothenberg EV. An Early T Cell Lineage Commitment Checkpoint Dependent on the Transcription Factor Bcl11b. Science (2010) 329(5987):89–93. 10.1126/science.1188989 20595614 PMC2935300

[84] Yu Y Wang C Clare S Wang J Lee SC Brandt C. The Transcription Factor Bcl11b is Specifically Expressed in Group 2 Innate Lymphoid Cells and is Essential for Their Development. J Exp Med (2015) 212(6):865–74. 10.1084/jem.20142318 PMC4451136 25964371

[85] Bando JK Colonna M. Innate Lymphoid Cell Function in the Context of Adaptive Immunity. Nat Immunol (2016) 17(7):783–9. 10.1038/ni.3484 PMC5156404 27328008

[86] Laurent J Bosco N Marche PN Ceredig R. New Insights Into the Proliferation and Differentiation of Early Mouse Thymocytes. Int Immunol (2004) 16(8):1069–80. 10.1093/intimm/dxh108 15197172

[87] Shinkai Y Rathbun G Lam KP Oltz EM Stewart V Mendelsohn M. Rag-2-deficient Mice Lack Mature Lymphocytes Owing to Inability to Initiate V(D)J Rearrangement. Cell (1992) 68(5):855–67. 10.1016/0092-8674(92)90029-c 1547487

[88] Mombaerts P Iacomini J Johnson RS Herrup K Tonegawa S Papaioannou VE. Rag-1-deficient Mice Have No Mature B and T Lymphocytes. Cell (1992) 68(5):869–77. 10.1016/0092-8674(92)90030-g 1547488

[89] Diamond RA Ward SB Owada-Makabe K Wang H Rothenberg EV. Different Developmental Arrest Points in RAG-2 -/- and SCID Thymocytes on Two Genetic Backgrounds: Developmental Choices and Cell Death Mechanisms Before TCR Gene Rearrangement. J Immunol (1997) 158(9):4052–64. 9126963

[90] Vaidya HJ Briones Leon A Blackburn CC. FOXN1 in Thymus Organogenesis and Development. Eur J Immunol (2016) 46(8):1826–37. 10.1002/eji.201545814 PMC4988515 27378598

[91] Roediger B Kyle R Yip KH Sumaria N Guy TV Kim BS. Cutaneous Immunosurveillance and Regulation of Inflammation by Group 2 Innate Lymphoid Cells. Nat Immunol (2013) 14(6):564–73. 10.1038/ni.2584 PMC4282745 23603794

[92] Wong SH Walker JA Jolin HE Drynan LF Hams E Camelo A. Transcription Factor RORalpha is Critical for Nuocyte Development. Nat Immunol (2012) 13(3):229–36. 10.1038/ni.2208 PMC3343633 22267218

[93] Zeis P Lian M Fan X Herman JS Hernandez DC Gentek R. In Situ Maturation and Tissue Adaptation of Type 2 Innate Lymphoid Cell Progenitors. Immunity (2020) 53(4):775–92.e9. 10.1016/j.immuni.2020.09.002 33002412 PMC7611573

[94] Huang Y Mao K Chen X Sun MA Kawabe T Li W. S1P-Dependent Interorgan Trafficking of Group 2 Innate Lymphoid Cells Supports Host Defense. Science (2018) 359(6371):114–9. 10.1126/science.aam5809 PMC6956613 29302015

[95] Teitelbaum SL Ross FP. Genetic Regulation of Osteoclast Development and Function. Nat Rev Genet (2003) 4(8):638–49. 10.1038/nrg1122 12897775

[96] Khodadadi L Cheng Q Radbruch A Hiepe F. The Maintenance of Memory Plasma Cells. Front Immunol (2019) 10:721. 10.3389/fimmu.2019.00721 31024553 PMC6464033

[97] Riedel R Addo R Ferreira-Gomes M Heinz GA Heinrich F Kummer J. Discrete Populations of Isotype-Switched Memory B Lymphocytes are Maintained in Murine Spleen and Bone Marrow. Nat Commun (2020) 11(1):2570. 10.1038/s41467-020-16464-6 32444631 PMC7244721

[98] Boberg E Johansson K Malmhall C Calven J Weidner J Radinger M. Interplay Between the IL-33/ST2 Axis and Bone Marrow ILC2s in Protease Allergen-Induced IL-5-Dependent Eosinophilia. Front Immunol (2020) 11:1058. 10.3389/fimmu.2020.01058 32582171 PMC7280539

[99] Boberg E Johansson K Malmhall C Weidner J Radinger M. House Dust Mite Induces Bone Marrow Il-33-Responsive ILC2s and TH Cells. Int J Mol Sci (2020) 21(11):3751. 10.3390/ijms21113751 PMC7312993 32466530

[100] Sudo T Motomura Y Okuzaki D Hasegawa T Yokota T Kikuta J. Group 2 Innate Lymphoid Cells Support Hematopoietic Recovery Under Stress Conditions. J Exp Med (2021) 218(5):e20200817. 10.1084/jem.20200817 PMC7941180 33666647

